# *Dicer* Is Required for Maintenance of Adult Pancreatic Acinar Cell Identity and Plays a Role in Kras-Driven Pancreatic Neoplasia

**DOI:** 10.1371/journal.pone.0113127

**Published:** 2014-11-18

**Authors:** Yue J. Wang, Florencia McAllister, Jennifer M. Bailey, Sherri-Gae Scott, Audrey M. Hendley, Steven D. Leach, Bidyut Ghosh

**Affiliations:** 1 The McKusick-Nathans Institute of Genetic Medicine, Johns Hopkins University School of Medicine, Baltimore, Maryland, United States of America; 2 The Department of Oncology, Johns Hopkins University School of Medicine, Baltimore, Maryland, United States of America; 3 The Department of Surgery, Johns Hopkins University School of Medicine, Baltimore, Maryland, United States of America; 4 The Graduate Program in Cellular and Molecular Medicine, Johns Hopkins University School of Medicine, Baltimore, Maryland, United States of America; University of Pittsburgh, United States of America

## Abstract

The role of miRNA processing in the maintenance of adult pancreatic acinar cell identity and during the initiation and progression of pancreatic neoplasia has not been studied in detail. In this work, we deleted *Dicer* specifically in adult pancreatic acinar cells, with or without simultaneous activation of oncogenic Kras. We found that *Dicer* is essential for the maintenance of acinar cell identity. Acinar cells lacking *Dicer* showed increased plasticity, as evidenced by loss of polarity, initiation of epithelial-to-mesenchymal transition (EMT) and acinar-to-ductal metaplasia (ADM). In the context of oncogenic Kras activation, the initiation of ADM and pancreatic intraepithelial neoplasia (PanIN) were both highly sensitive to *Dicer* gene dosage. Homozygous *Dicer* deletion accelerated the formation of ADM but not PanIN. In contrast, heterozygous *Dicer* deletion accelerated PanIN initiation, revealing complex roles for *Dicer* in the regulation of both normal and neoplastic pancreatic epithelial identity.

## Introduction

The microRNA pathway is a critical regulator of gene expression, and Dicer, an RNase III-containing enzyme, is a central component of the microRNA (miRNA) processing machinery. Deregulation of miRNAs has been implicated in a variety of biological process including cancer [1], [2]. In mice, global loss of *Dicer* leads to lethality before embryonic day 7.5 [3]. Conditional inactivation of *Dicer* in numerous cell types from tissues such as the immune system [4], retina [3], cortex, hippocampus [5] and ovary [6], has proven the essential function of *Dicer* in a number of tissue-specific contexts.

Previous work has shown that miRNA levels fluctuate during cancer initiation and progression. Both tumor suppressor and oncogenic miRNAs have been described [7], [8]. In general, tumor cells display a global downregulation of miRNA expression [9]. Recently, several reports have indicated that *Dicer* may function as a haploinsufficient tumor suppressor, as heterozygosity for *Dicer* enhances tumor development, whereas enforced *Dicer* homozygous deletion causes inhibition of tumorigenesis in certain tumor models [10], [11], [12].

Recent publications have shown that pancreatic acinar cells display unexpected plasticity, and are capable of undergoing dramatic changes in differentiation in the settings of both chronic pancreatitis and pancreatic cancer. Lineage tracing approaches have provided evidence that acinar cells in the adult pancreas have the potential to transdifferentiate into metaplastic ductal cells [13]. This acinar-to-ductal metaplasia (ADM) event precedes the formation of pancreatic intraepithelial neoplasia (PanIN) [14], [15], [16], [17]. Acinar cells have similarly been demonstrated to be an effective cell-of-origin for *Kras^G12D^*-induced PanIN and pancreatic ductal adenocarcinoma (PDAC) [18], [19], [20].

Deletion of *Dicer* at the onset of pancreatic development results in defects in all pancreatic lineages [21], while loss of *Dicer* in the pancreas at a late gestational stage (E18.5) results in defects in acinar cell differentiation and morphogenesis [22]. Interestingly, the adult pancreas seems to be extremely sensitive to changes in the abundance of *Dicer* [23]. In the *Dicer* hypomorphic mouse created by Morita et al [23], *Dicer* expression was reduced to 20% in all tissues and the only observed abnormality was in the adult pancreas where multinucleated cells were found and both of the endocrine and exocrine cells had unusual organization.

While previous findings suggest important roles for *Dicer* during pancreatic development, its role in adult acinar cells has not yet been assessed. In the present study, we triggered loss of *Dicer* in the adult exocrine pancreas with or without simultaneous activation of oncogenic *Kras^G12D^*. In normal pancreata, deletion of both copies of *Dicer* resulted in the progressive loss of differentiated acinar cells, associated with the initiation of epithelial-to-mesenchymal transition (EMT). These changes were additionally accompanied by ADM and pancreatic fibrosis. In concert with *Kras* ^G12D^ activation, homozygous *Dicer* deletion accelerated ADM formation, but not PanIN initiation. In contrast, PanIN formation was enhanced in the setting of *Dicer* haploinsufficiency. Regardless of genotype, PanIN lesions were observed to have retained Dicer expression. Thus, in addition to being required for the maintenance of acinar cell identity, fractional changes in Dicer gene activity may have significant impact on the pancreatic response to oncogenic stimuli.

## Materials and Methods

### Mouse lines

*Dicer^fl/fl^* mice were crossed with *Mist1^CreERT2/+^* mice (gift from Stephen F. Konieczny**,** University of Indiana). An additional *lox-stop-lox Rosa26 YFP* allele (hereafter referred to as *LSL-YFP*) was incorporated as a lineage marker. The resulting *Mist1^CreERT2/+^; LSL-YFP*; *Dicer^fl/wt^* mice were further intercrossed or outcrossed onto a conditional *LSL-Kras^G12D^* allele, as previously described [18]. Mice were euthanized by cervical dislocation under Isoflurane anesthesia. There was no surgery performed and animals exhibiting discomfort were euthanized. All procedures were performed under the approval of the Johns Hopkins University School of Medicine Animal Care and Use Committee guidelines (IACUC, protocol number M012M353).

### Tamoxifen induced Dicer deletion

Induction of CreERT2 activity was initiated at 6–8 weeks of age by administering 5 mg of tamoxifen (Sigma, 10540-29-1) for 3 consecutive days via intra-peritoneal (IP) injections. Control littermates were injected with corn oil alone.

### Pancreatic acinar isolation and in vitro culture

Acinar units were isolated as described previously [24]. Briefly, mouse pancreata were perfused from common bile duct with 0.375 mg/ml Collagenase-P (Roche, 11213857001) in Hank’s balanced salt solution (HBSS). Subsequently, pancreata were harvested and incubated in 37°C water bath for 11 mins. Following multiple washes with HBSS and 5% FBS, hand-picked acini were cultured in RPMI 1640 supplemented with 10% fetal bovine serum (FBS) (Invitrogen), soybean trypsin inhibitor (Sigma) and antibiotics in low attachment 24 well plates (Corning). Acini were treated with either DMSO or with 4-hydroxytamoxifen (Sigma, H7904) at 20 ng/ml for 5 consecutive days to induce Cre-mediated deletion of *Dicer* in vitro.

### Immunohistochemistry and immunofluorescence

Pancreata were harvested at indicated time points and were fixed in 4% paraformaldehyde, followed by standard paraffin or OCT embedding. Tissue was then cut into 5–10 µM sections. Incubations with primary antibodies were performed overnight at 4°C using standard techniques in PBS containing 0.2% Triton and 10% FBS using the following antibodies or probes: rat anti-CD49f (BD Biosciences 555734, 1∶100), phalloidin (Invitrogen R415, 1∶500), rat anti-Ecad (Invitrogen 131900, 1∶200), rabbit anti-Ki67 (Abcam Ab16667, 1∶50), rabbit anti-Sox9 (Millipore ab5535, 1∶200), chicken anti-Vimentin (Millipore ab5733, 1∶500), rat anti-Ncad (Abcam Ab12221, 1∶200), rat anti-EpCam (Biolegend, 118214, 1∶100), rabbit anti-Amalyse (Sigma A8273, 1∶500), DBA (Vector Labs AS2034, 1∶200), rabbit anti-Dicer (Clonegene CG031, 1∶1000). For immunohistochemistry, HRP conjugated secondary antibodies were obtained from Vector Labs (MP-7401). 3-3-Diaminobenzidine tetrahydrochloride (Vector Labs) was used as a chromogen. Bright-field images were acquired using an Olympus BX40 light microscope. For immunofluorescence, secondary antibodies were obtained from Jackson ImmunoResearch and used at 1∶300 dilution. Samples were mounted with Fluorescence Mounting Medium (Dako, S3023). Slides were imaged on the Nikon A1 confocal microscope system. ImageJ was used to quantify the areas of specific lesions. Student t-test was performed for compare different groups.

## Results

### Dicer knockout in mature pancreatic acinar cells

**Figure 1:**
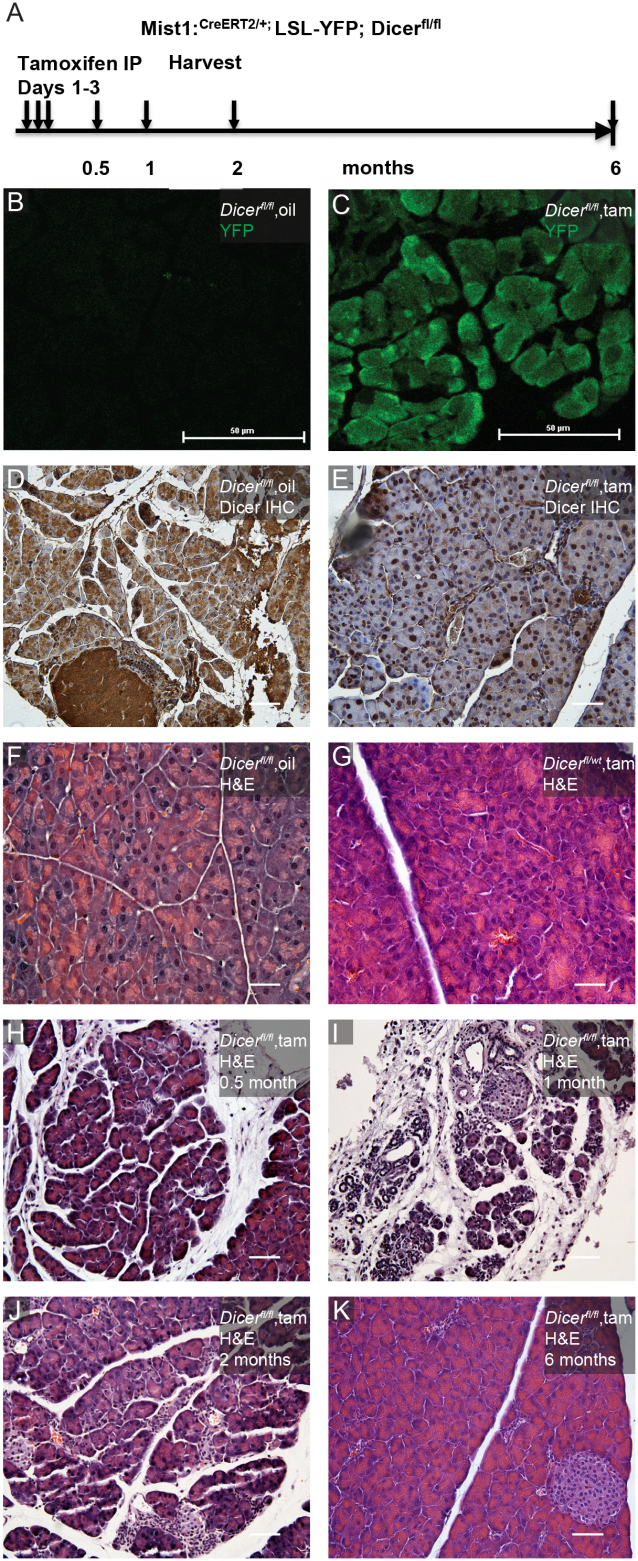
Deletion of *Dicer* alters histology in exocrine pancreas. (A) Experimental regimen. Tamoxifen injection was performed on 6–8 weeks old mice. (B) Pancreas 2 months after treatment with corn oil has no YFP expression. (C) Widespread Cre-mediated recombination is shown by YFP expression 2 month following tamoxifen induction. (D, E) Immunohistochemistry for Dicer on *Mist-Cre^ERT2^; LSL-YFP; Dicer^fl/fl^* mice 2 month after treatment with corn oil only (D) or those treated with tamoxifen (E). There is almost complete loss of cytoplasmic Dicer signal in the acinar cells in tamoxifen treated tissue. Residual nuclear signal likely represents non-specific antibody labeling. (F–K) H & E staining showing the time-course of histologic change after tamoxifen administration. (F) *Dicer^fl/fl^* Oil control. (G) *Dicer^fl/wt^*. Tissue is morphologically indistinguishable from oil control. (H) *Dicer^fl/fl^* 0.5 month after tamoxifen injection. (I) *Dicer^fl/fl^* 1 month after tamoxifen injection. Tissue changes are most dramatic at this time point. (J) 2 months. (K) 6 months. At this time point, pancreas from Dicer knockout mice is nondistinguishable with wildtype. Scale bars depict 50 microns.

In order to investigate the role of *Dicer* in the maintenance of adult acinar cell differentiation, we generated *Mist1^CreERT2^; Dicer^fl/fl^; LSL-YFP* mice [18]. Tamoxifen was administered to 8-week-old adult mice via intraperitoneal injection. We followed morphological changes in pancreata up to 6 months after tamoxifen injection (Fig. 1A). Successful Cre-mediated recombination could be visualized by the expression of YFP in more than 90% of the acini (Fig. 1B, C) and the expression of YFP was highly correlated with the elimination of Dicer protein, as assessed by Dicer immunohistochemistry (Fig. 1D, E). During the time course of this study, the most extensive pancreatic abnormalities were observed at one month following the deletion of *Dicer* (Fig. 1F–K, Fig. S1). In some cases, pancreatic lobes were characterized by extreme acinar cell atrophy, with only remnants of ductal like structures left behind (Fig. 1I). This was accompanied by a prominent inflammatory infiltrate (Fig. 1I). In the most severe scenario, areas of fibrosis were observed to replace the majority of normal pancreatic acinar tissue (Fig. S1A, B). By six months post tamoxifen administration, the histology of pancreata resumed normality with little sign of tissue damage (Fig. 1K). Noticeably, at six months after the deletion *of Dicer*, regions of YFP expression became more sporadic (Fig. S1, Fig. S2). The histology of the pancreata from Mist1^creERT2^; *Dicer* ^fl/wt^ animals remained normal across all time points (Fig. 1G).

### Dicer is required for the maintenance of mature acinar cell identity

**Figure 2:**
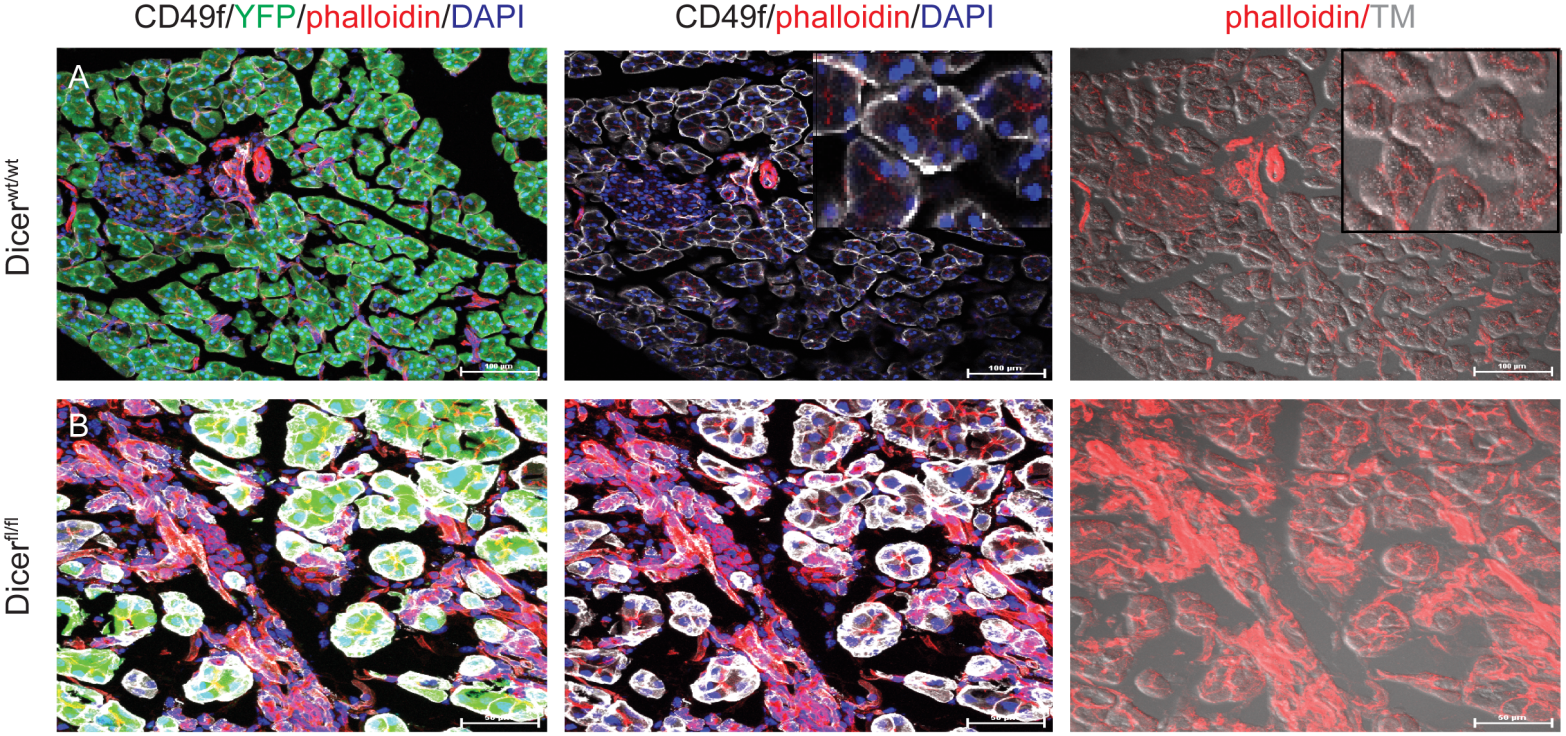
Deletion of *Dicer* induces loss of polarity in acinar cells following Dicer deletion. (A) Epithelial cell polarity marker CD49f (white) is expressed basally, while phalloidin labeling (red) is observed apically in control *Mist-Cre^ERT2^; LSL-YFP; Dicer^wt/wt^* pancreas following tamoxifen treatment. (B) In the *Mist-Cre^ERT2^; LSL-YFP; Dicer^fl/fl^* mice, *Dicer* deletion leads to translocation of CD49f in the lateral membrane, and loss of apical phalloidin labeling. Cytoplasmic expression of YFP is shown in green as a surrogate marker of Cre-mediated recombination. Nuclei are stained with DAPI (blue).

We sought to characterize changes occurring following acinar cell-specific *Dicer* deletion in additional detail. We used two markers of cell polarity, CD49f (integrin subunit α6) and phalloidin. CD49f forms a heterodimer with integrin β1 subunit and is present on the basal membrane of pancreatic acini [25]; while phalloidin stains the actin web concentrated at the apical side of acinar cells. Using immunofluorescent staining, we found that these polarity markers were mislocalized in the acinar cells of the *Dicer* knockout. CD49f translocated to the lateral membrane (Fig. 2) and the expression level of CD49f was significantly increased compared with control acini. Phalloidin showed a diffuse pattern of staining in the *Dicer* deleted animals, rather than labeling apically-oriented actin filaments (Fig. 2), indicating a loss of cell polarity.

**Figure 3:**
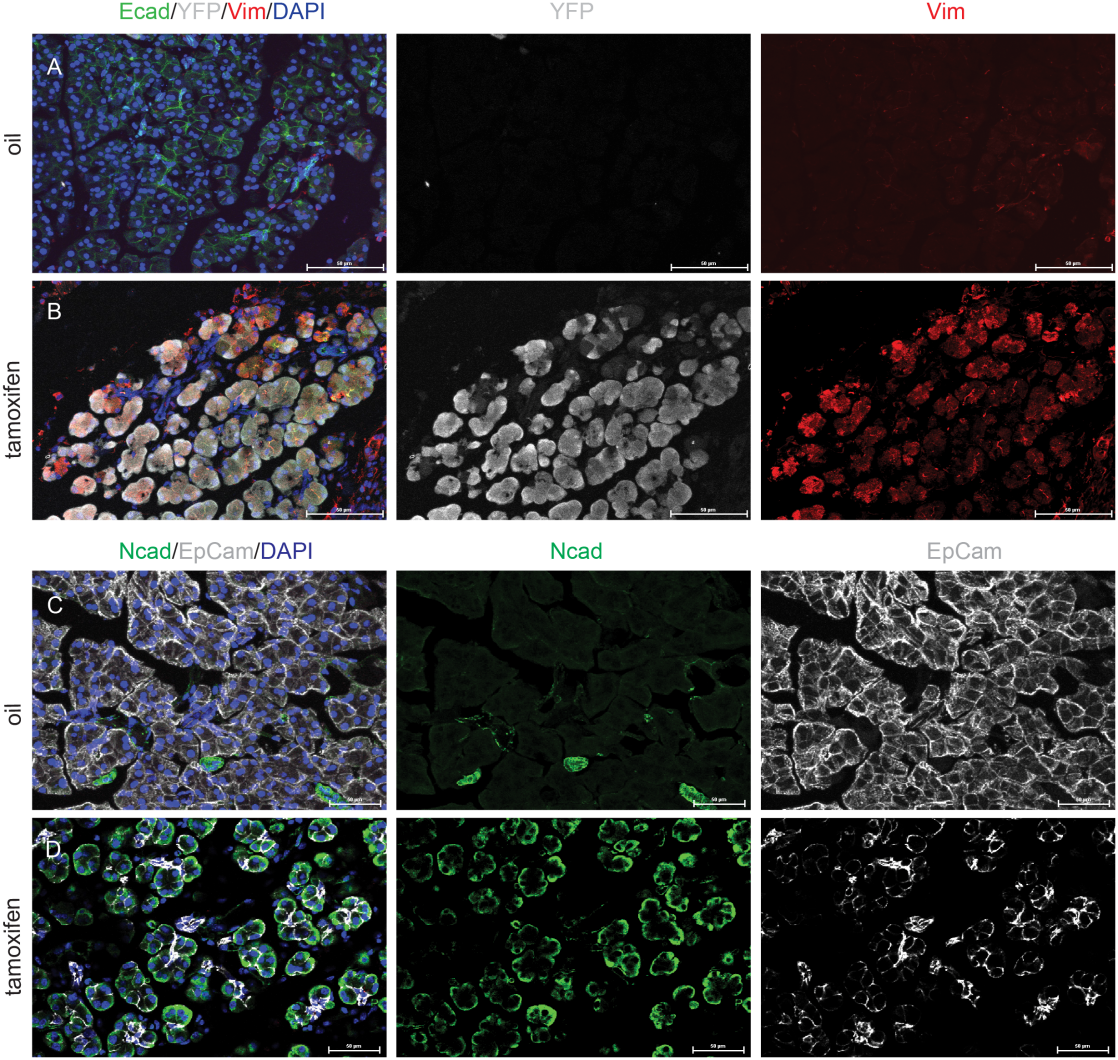
Adult acinar cells undergo epithelial to mesenchymal transition. (A) Pancreata from oil injected *Mist-Cre^ERT2^; LSL-YFP; Dicer^fl/fl^* mice do not express YFP (white) or Vimentin (red). Ecad (green) outlines acinar cells. (B) In tamoxifen treated *Mist-CreERT2; LSL-YFP; Dicer^fl/fl^* mice, acinar cells show co-localization of YFP and Vimentin. Ecad expression is reduced. (C, D) Representative immunofluorescent staining for the mesenchymal marker Ncad (green). (C) In oil treated control pancreas, acini lack any Ncad expression. (D) In tamoxifen treated *Dicer^fl/fl^* pancreas, there is membrane expression of Ncad. Note that there is simultaneous loss of EpCam expression (white), in *Dicer* deleted acini. Nuclei are stained with DAPI (blue).

Following *Dicer* deletion, pancreatic acinar cells also lost their classic rosette-like epithelial organization (Fig. 2B, Fig. 3B, D). This phenotype was similar to what was observed by Morita et al [23] in the *Dicer* hypomorphic pancreas. In addition, acinar cells lacking Dicer developed a partial mesenchymal phenotype. This process of EMT was documented by decreased expression of the epithelial marker Ecad, EpCam and increased expression of the mesenchymal markers Vimentin and Ncad (Fig. 3).

**Figure 4:**
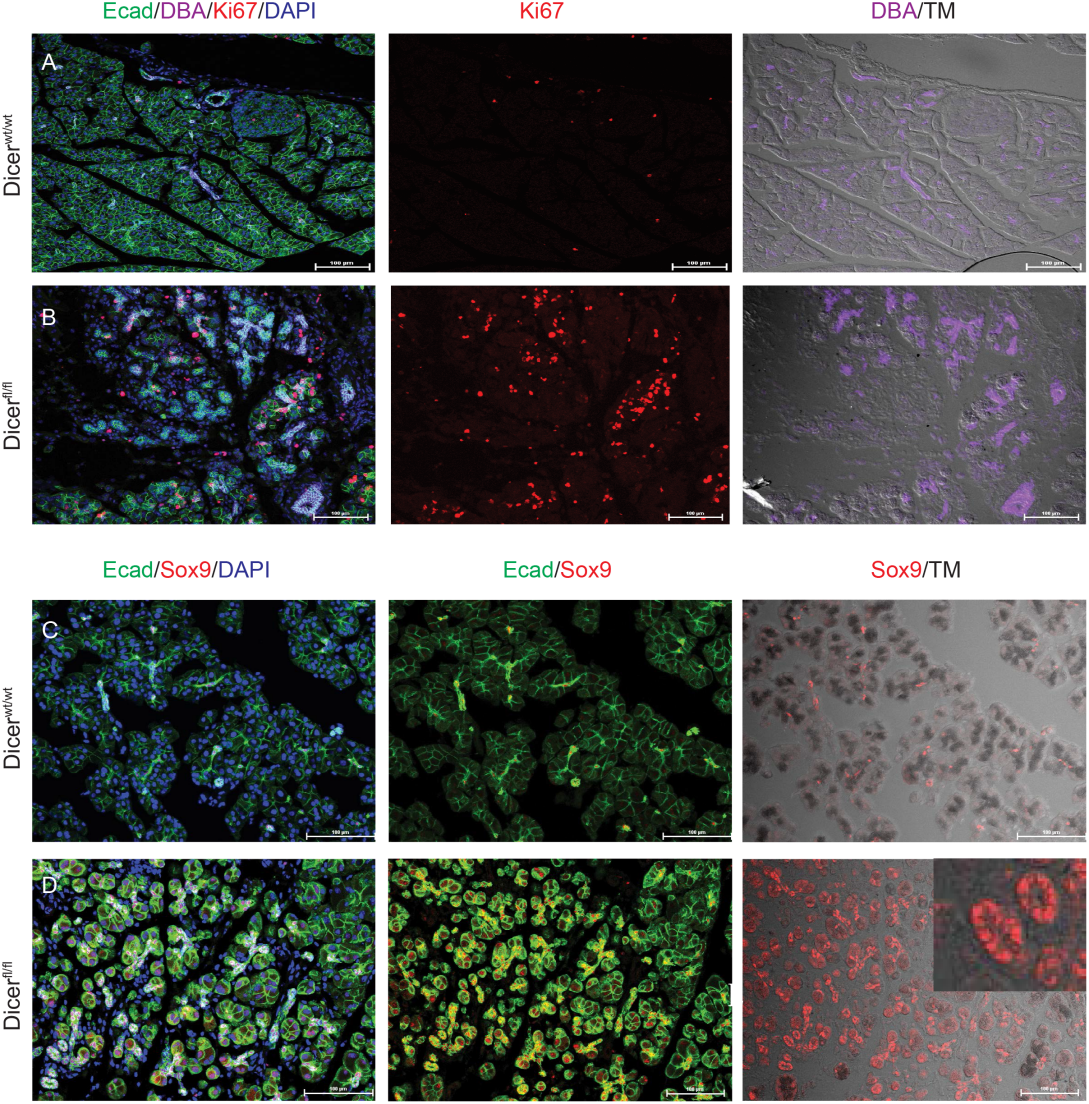
*Dicer* deletion leads to upregulation of ductal markers in the acinar population. (A, B) Pancreatic ductal marker Dolichos Biflorus Agglutinin (DBA) (purple) is upregulated along with cell proliferation marker Ki67 (red) in the *Mist-Cre^ERT2^; Dicer^fl/fl^* mice following tamoxifen injection (B) compared to control *Mist-Cre^ERT2^; Dicer^wt/wt^* pancreata (A). (C, D) Sox9 (red), a duct-specific transcription factor, is also upregulated in acinar cells and in ADM lesions in *Mist-Cre^ERT2^; Dicer^fl/fl^* pancreata (D), while Sox9 is expressed only in ductal epithelial cells in control *Mist-Cre^ERT2^; Dicer^wt/wt^* pancreata (C). Epithelial marker Ecad is in green and nuclei are stained with DAPI (blue).

Furthermore, in the absence of *Dicer*, some of the acinar cells showed evidence of ADM (Fig. 4). This was indicated by appearance of the YFP positive lineage marker in some of the duct-like structures, which were labelled by the lectin Dolichos Biflorus Agglutinin (DBA) as well as an antibody against Sox9 (Fig. 4, Fig. S1C, D). Following *Dicer* deletion, proliferative responses were observed in both acinar and nonacinar cells, presumably as a result of signals from pancreatic injury (Fig. 4A, B). An extensive quantification of Ki67 positive cells following tamoxifen administration revealed that maximum cellular proliferation occurred in the acinar cells at one month after loss of *Dicer*, with less frequent proliferation observed in other components of pancreas including ducts, islets and stroma (Fig. S2).

In summary, we observed a major identity shift in the acinar population upon homozygous *Dicer* knockout. Acinar cells lost apical-basal polarity and transdifferentiated into ductal or mesenchymal cell types, suggesting that Dicer is required for the maintenance of acinar cell differentiation.

### In vitro acinar cultures confirm a cell autonomous effect of Dicer deletion

**Figure 5:**
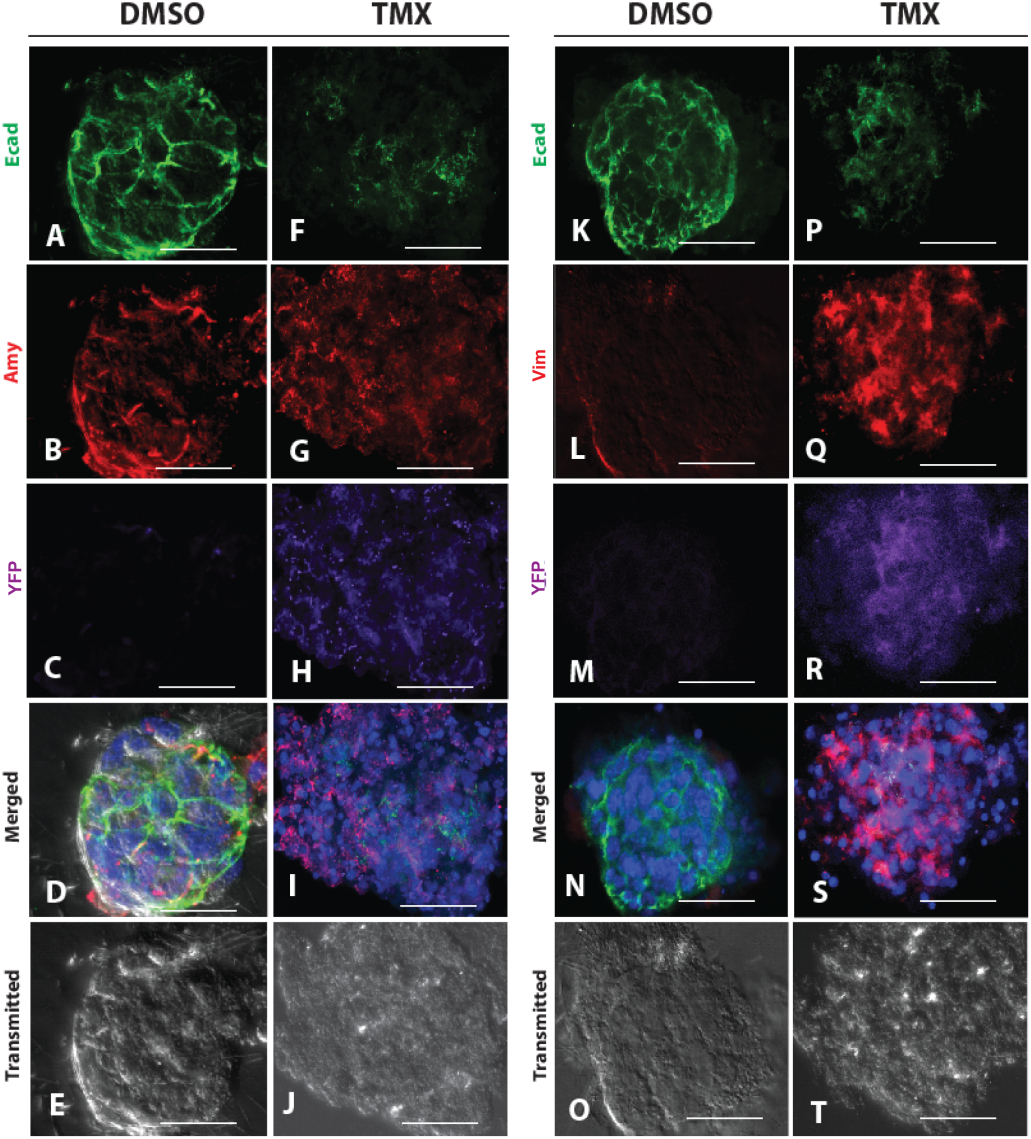
*In vitro* deletion of *Dicer* in pancreatic acinar cells results in initiation of EMT. (A–E) and (K–O) depict multi-channel fluorescent imaging of two representative pancreatic acini independently harvested from *Mist1^CreERT2/+^*; *LSL-YFP; Dicer^fl/fl^* mice and treated with DMSO control. (F–J) and (P–T) depict multi-channel fluorescent imaging of two representative pancreatic acini isolated from *Mist1^CreERT2/+^*; *LSL-YFP; Dicer^fl/fl^* mice and treated with tamoxifen (TMX) to induce Cre activity. (A–J), Immunostaining of isolated acinus with Amylase (red), Ecad (green) and GFP (purple) There is no change in Amylase expression following 5 days incubation with either DMSO control (A–E) or tamoxifen treatment (F–J). But significant down-regulation of Ecad can be observed. (K–T), Immunostaining of isolated acini with Vimentin (red), Ecad (green) and YFP (purple). Down-regulation of Ecad is accompanied by activation of Vimentin in tamoxifen-treated cells (P–T), compared to DMSO control (K–O). Activation of YFP in (H) and (R) document tamoxifen-induced Cre activity.

In the *Dicer* knockout pancreas, an activated inflammatory response is observed (Fig. 1). Based on the fact that morphological changes in pancreatic acinar cells can be induced by pancreatic inflammation alone [13], [26], we endeavored to assess whether the phenotypes we observed after *Dicer* deletion in acinar cells were a primary effect of *Dicer* knockout or a secondary effect of tissue injury. For these studies, we utilized an *in vitro* acinar culture system [24]. In this setup, the macrophages, neutrophils and other components of the immune system were absent. Isolated acini from *Mist1^CreERT2/+^; LSL-YFP; Dicer^fl/fl^* mice were handpicked and cultured in the presence of 4-hydroxytamoxifen (4-OH) or DMSO control. Acini incubated with 4-OH showed down-regulation of Ecad (Fig. 5A, K vs. 5F, P) and up-regulation of Vimentin (Fig. 5L vs. 5Q); similar to what we observed *in vivo* (Fig. 3). Acini incubated with DMSO control maintained their epithelial identity (Fig. 5K, L, M, N, O). In these experiments, YFP expression following 4-OH treated acini was used as a surrogate marker for successful deletion of *Dicer* (Fig. 5C, H, M, R). Amalyse staining was used to confirm the acinar cell type (Fig. 5B, G). These studies demonstrate a cell autonomous requirement for *Dicer* in the maintenance of acinar cell identity.

### *Dicer* haploinsufficiency enhances PanIN formation in an oncogene-dependent manner

**Figure 6:**
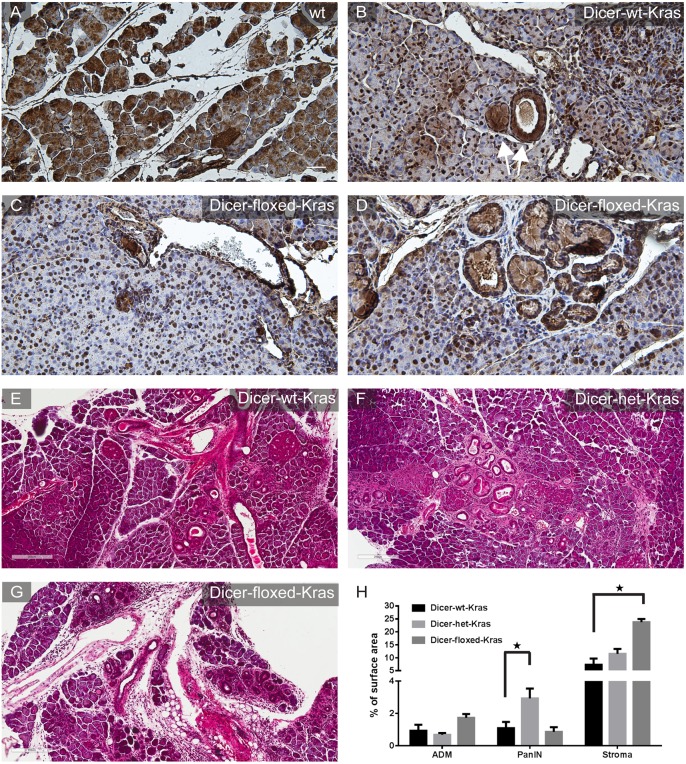
Kras-driven pancreatic lesions are sensitive to Dicer gene dosage. (A–D) Brown staining indicates immunohistochemical labeling for Dicer (hemotoxylin counterstain, blue). (A) Wildtype pancreas. There is strong cytoplasmic labeling for Dicer in acinar cells. (B) *Dicer-wt-Kras*. Dicer is down-regulated in acinar cells following oncogenic Kras activation. Notice that PanIN lesions (arrows) have higher Dicer expression. (C) *Dicer-floxed-Kras*. Residual nuclear signals likely represent non-specific antibody labeling. (D) In the *Dicer-floxed-Kras* pancreata, areas of ADM retain higher levels Dicer expression. Normal ductal epithelium, stromal cells and endocrine cells (lower right) also show normal expression of Dicer. E–G, H&E labeling demonstrates representative histology (E) *Dicer-wt-Kras*, (F) *Dicer-het-Kras* and (G) *Dicer-floxed-Kras* pancreata. (H) Quantification of surface area occupied by stromal infiltrate, ADM and PanIN. n>5 for each genotype. Error bars indicate standard error. Star denotes comparisons with p-value<0.05.

Loss of *Dicer* in normal adult pancreatic acinar tissue resulted in the initiation of ADM, which is a proposed precursor of PanIN [16], [27], [17]. We therefore sought to determine whether loss of *Dicer* would facilitate PanIN initiation. Recent studies have shown that the *Mist1^CreERT2/+^; LSL-Kras^G12D^* mouse model fully recapitulates the initiation and progression of early human pancreatic neoplasia [18]. To test the function of Dicer in the context of oncogenic Kras-driven pancreatic neoplasia, we crossed *Dicer^fl/fl^* mice onto the *Mist1^CreERT2/+^; LSL-Kras^G12D^* mice. We generated *Mist1^CreERT2/+^; LSL-Kras^G12D^*; *Dicer^fl/fl^* (*Dicer-floxed-Kras*) and *Mist1^CreERT2/+^; LSL-Kras^G12D^*; *Dicer^fl/+^* (*Dicer-het-Kras*) mice, and compared them with *Mist1^CreERT2/+^; LSL-Kras^G12D^*; *Dicer^wt/wt^* (*Dicer-wt-Kras*) mice. Interestingly, immunohistochemistry for Dicer in the pancreata of *Dicer-wt-Kras* mice demonstrated a tissue-wide decrease of Dicer protein (Fig. 6A vs B). This observation is in line with the hypothesis that the global down-regulation of miRNA expression in tumors may be driven by alterations in the expression levels or activity of Dicer [28]. Notably, in the same sections, PanIN lesions displayed higher levels of *Dicer* expression (Fig. 6B, arrows). In the *Dicer-floxed-Kras* mice, there was an almost complete loss of cytoplasmic Dicer protein in the acinar compartment, although some nuclear Dicer staining remained, likely representing nonspecific signal (Fig. 6C). Strikingly, the vast majority of metaplastic and neoplastic lesions preserved a moderate level of Dicer protein (Fig. 6D), suggesting selective pressure to retain at least some level of Dicer expression during the initiation of pancreatic neoplasia. This is consistent with recent work showing certain types of tumors select against full loss of Dicer function [10].

We quantified the pancreatic phenotypic abnormalities, including ADM and PanIN, in both the *Dicer-floxed-Kras* and *Dicer-het-Kras* mice. We observed that *Dicer-het-Kras* mice developed PanINs at earlier time points and at much higher frequency than the *Dicer-floxed-Kras* mice and the *Dicer-wt-Kras* mice in the same cohort (Fig. 6E–H). At the two-month time point, *Dicer-het-Kras* mice had PanIN comprising approximately 4% of total pancreatic surface area, compared to the 0.8% in *Dicer-floxed-Kras* mice and 1% in *Dicer-wt–Kras* mice (Fig. 6H). In contrast, *Dicer-floxed-Kras* mice have an increase in ADM compared with *Dicer-het-Kras* and *Dicer-wt-Kras* pancreata. (Fig. 6H). This is consistent with our previous assumption, indicating that *Dicer* loss accelerates the formation of ADM under conditions of Kras activation, but in the absence of Dicer these ADM lesions are unable to further progress to PanIN.

## Discussion

Dicer is a master regulator of the miRNA pathway. The deletion of Dicer leads to loss of mature miRNAs [29], [30]. To assess the requirement of miRNAs in adult acinar tissue, we combined a floxed *Dicer* allele with an exocrine pancreas specific, tamoxifen-inducible Cre driver, *Mist1^CreERT2^* [18]. In this report we show that *Dicer* is essential for the maintenance of normal acinar cell identity, with homozygous Dicer deletion resulting in loss of acinar cell polarity, and the induction of both ADM and EMT. When combined with activation of oncogenic Kras, homozygous Dicer deletion leads to accelerated ADM formation but no change in the rate of PanIN initiation or progression. We also present evidence that PanIN lesions select against complete loss of Dicer, and that Dicer haploinsufficiency accelerates PanIN initiation.

In non-neoplastic pancreas, histologic changes associated with conditional Dicer deletion were manifested within 72 hours following tamoxifen injection (data not shown). These cellular responses included loss of cell polarity and the induction of EMT and ADM, indicating an overall increase in cellular plasticity. *In vitro* induction of Dicer deletion in isolated pancreatic acini confirmed that these changes represented a cell autonomous effect of *Dicer* loss. In the *in vivo* setting, we did observe a robust inflammatory response after *Dicer* deletion (Fig. 1). In fact, some of the hyperplastic ductal-like structure we observed were not of apparent acinar cell origin, based on the absence of a heritable lineage marker (Fig. S1). While we cannot entirely rule out the possibility that the absence of lineage marker expression is due to mosaic Cre activity, these structures may have arisen from ductal or other cell populations as a non-cell autonomous response to acinar cell injury.

In the absence of Kras activation, acinar cells eventually regenerated following widespread *Dicer* deletion. We observed increasingly patchy YFP expression in the regenerated pancreas (Fig. S1, Fig. S2). We believed that the expanding pancreatic exocrine tissue originated from acinar cells that had escaped *Dicer* deletion [13], [31]. However, we cannot exclude other possibilities such as a transdifferentiated and/or a progenitor cell population contributing to the regenerated pancreata [32]. In fact, we observed a general proliferative response even in non-acinar tissue after *Dicer* deletion (Fig. 4B, Fig. S2).

When accompanied by Kras activation, ADM lesions persisted and progressed. Based on persistent immunohistochemical labeling, these ADM and PanIN lesions seemed to select against complete loss of *Dicer*. This observation is in line with multiple cancer models in which enforced *Dicer* deletion cause inhibition of tumorigenesis, and tumors from *Dicer* ^fl/fl^ animals typically maintain one functional *Dicer* allele [10], [11], [12], [33]. One potential limitation of our study is that both the floxed *Dicer* alleles and *LSL-Kras^G12D^* allele were recombined simultaneously in response to tamoxifen administration. One possibility is that during different stages of pancreatic tumor initiation and progression, there are divergent requirements for different levels of Dicer activity. Further studies that temporally separate the *Dicer* deletion and *Kras* activation events, as might be achieved using a tetracycline-regulated system [34], [35], are needed to further investigate time- and tumor stage-dependent influences of Dicer.

While this manuscript was under preparation, a study by Morris et al was published describing the role of Dicer in the regulation of Kras-mediated ADM and PanIN [36]. This study reported that loss of *Dicer* compromised acinar identity and promoted Kras driven ADM. At the same time, *Dicer* loss did not accelerate PanIN or PDAC development. Overall, their findings are comparable to our results. The major differences between this paper and the current study involve the use of different Cre driver lines. In the manuscript by Morris and colleagues, a *Pdx1:Cre* line was utilized in which Cre activity is initiated in multi-lineage embryonic pancreatic progenitors at E12 [37]. In this setting, *Dicer* is conditionally deleted in all pancreatic epithelial lineages, while in our study, we have interrogated the function of *Dicer* specifically in adult acinar cells. Using this approach, we demonstrate that *Dicer* loss restricted to adult acinar cells is sufficient to induce an ADM reprogramming process, as characterized by the induction of DBA and Sox9 expression. A recent study demonstrated that the upregulation of Sox9 in the acinar population is an early tumor-initiating event [38]. Thus, miRNA deregulation in the acinar compartment may induce an early cellular change that renders acinar cells more prone to additional oncogenic stimuli, including Kras activation.

In conclusion, we have established that Dicer is essential for the maintenance of acinar cell identity in the adult post-differentiated exocrine pancreas. Different levels of Dicer may be required at different stages of Kras-driven pancreatic tumorigenesis. Further work is needed to examine the functions of individual microRNAs and the time-dependent role of Dicer during pancreatic cancer initiation and progression.

## Supporting Information

Figure S1 ***Dicer*** **deletion leads to pancreatic fibrosis.** (A–B) Trichrome staining shows fibrosis in the pancreas of 1 month post tamoxifen treatment. (A) Oil control. (B) 1 month post tamoxifen. There is severe phenotype with partial loss of acinar mass and sclerotic tissue. (C) 6 months after tamoxifen administration, YFP expression (green) becomes patchy. Some of the acinar units resemble ductal like structures as shown by Ecad (white) staining. Arrow points to one ductal like structure that is positive for YFP. Arrowheads point to several ductal like structures that are negative for YFP. (D) Progression of histologic changes following tamoxifen treatment. The most extensive changes are evident at one month post-tamoxifen. By 6 months, the pancreas is almost completely regenerated.
(TIF)

Figure S2 **Tissue-wide proliferation response after** ***Dicer*** **deletion.** Ki67 (red) labels proliferating cells in pancreata (A) 75 h, (B) 2 weeks, (C) 1 months, (D) 2 months, (E) 6 months post tamoxifen administration. In (A) Ecad is shown in green, DBA in white and DAPI in blue. In B–E, YFP (green) labels cell lineages in which Cre-mediated recombination has occurred. Note relative expansion of YFP-negative regions at 2 months and 6 months post-tamoxifen. Sections are co-stained with DBA (white) and DAPI (blue). Quantification of Ki67-positive cells per high power field is shown in (F).
(TIF)
