# Supplementary figures and images for: Dicer Is Required for Maintenance of Adult Pancreatic Acinar Cell Identity and Plays a Role in Kras-Driven Pancreatic Neoplasia
Source: PLoS One. 2014 Nov 18;9(11):e113127. doi: 10.1371/journal.pone.0113127 (PMC4236134; doi:10.1371/journal.pone.0113127)

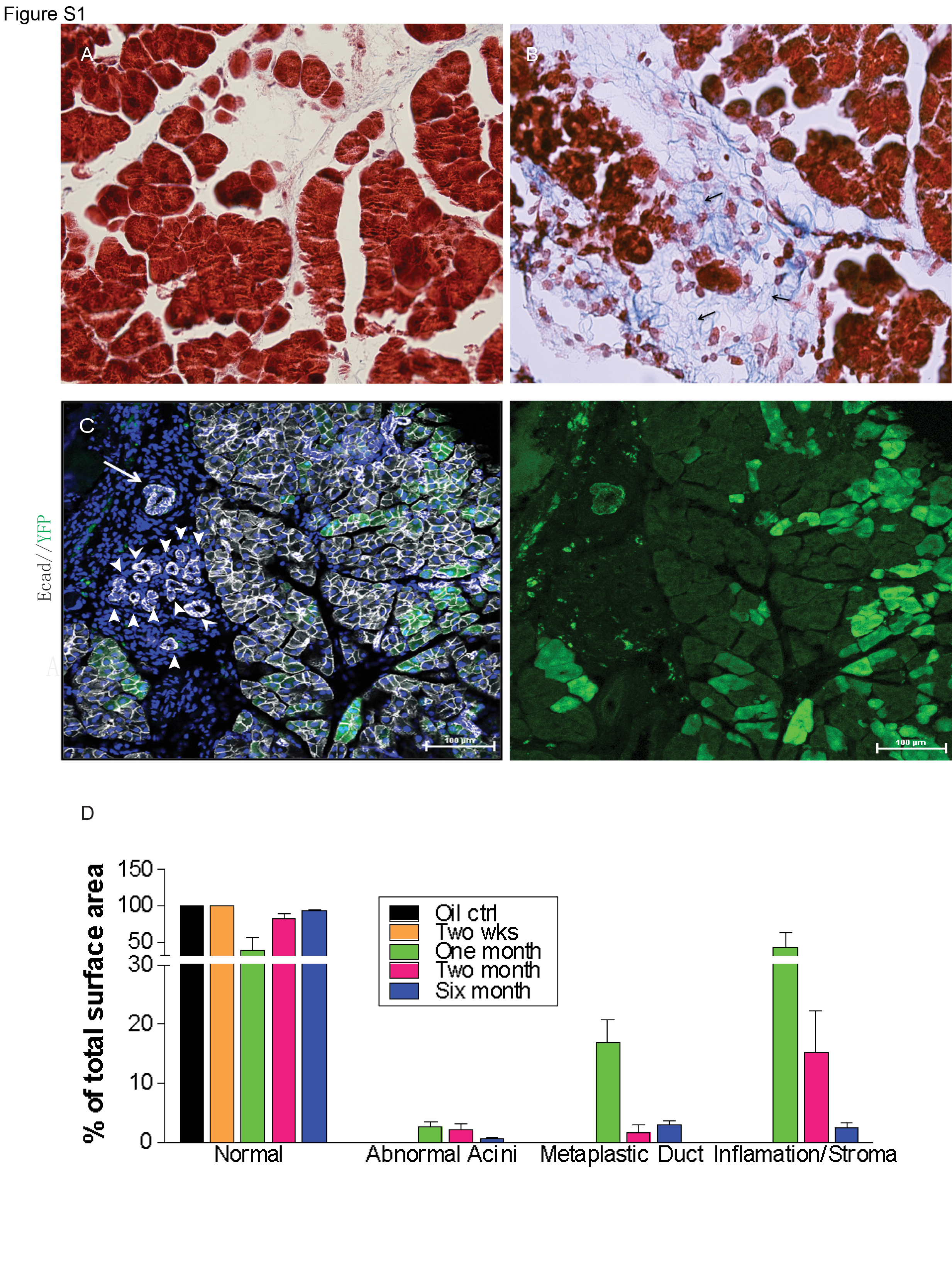

Supplement: Figure S1 — Dicer deletion leads to pancreatic fibrosis. (A–B) Trichrome staining shows fibrosis in the pancreas of 1 month post tamoxifen treatment. (A) Oil control. (B) 1 month post tamoxifen. There is severe phenotype with partial loss of acinar mass and sclerotic tissue. (C) 6 months after tamoxifen administration, YFP expression (green) becomes patchy. Some of the acinar units resemble ductal like structures as shown by Ecad (white) staining. Arrow points to one ductal like structure that is positive for YFP. Arrowheads point to several ductal like structures that are negative for YFP. (D) Progression of histologic changes following tamoxifen treatment. The most extensive changes are evident at one month post-tamoxifen. By 6 months, the pancreas is almost completely regenerated. (TIF) [file pone.0113127.s001.tif]

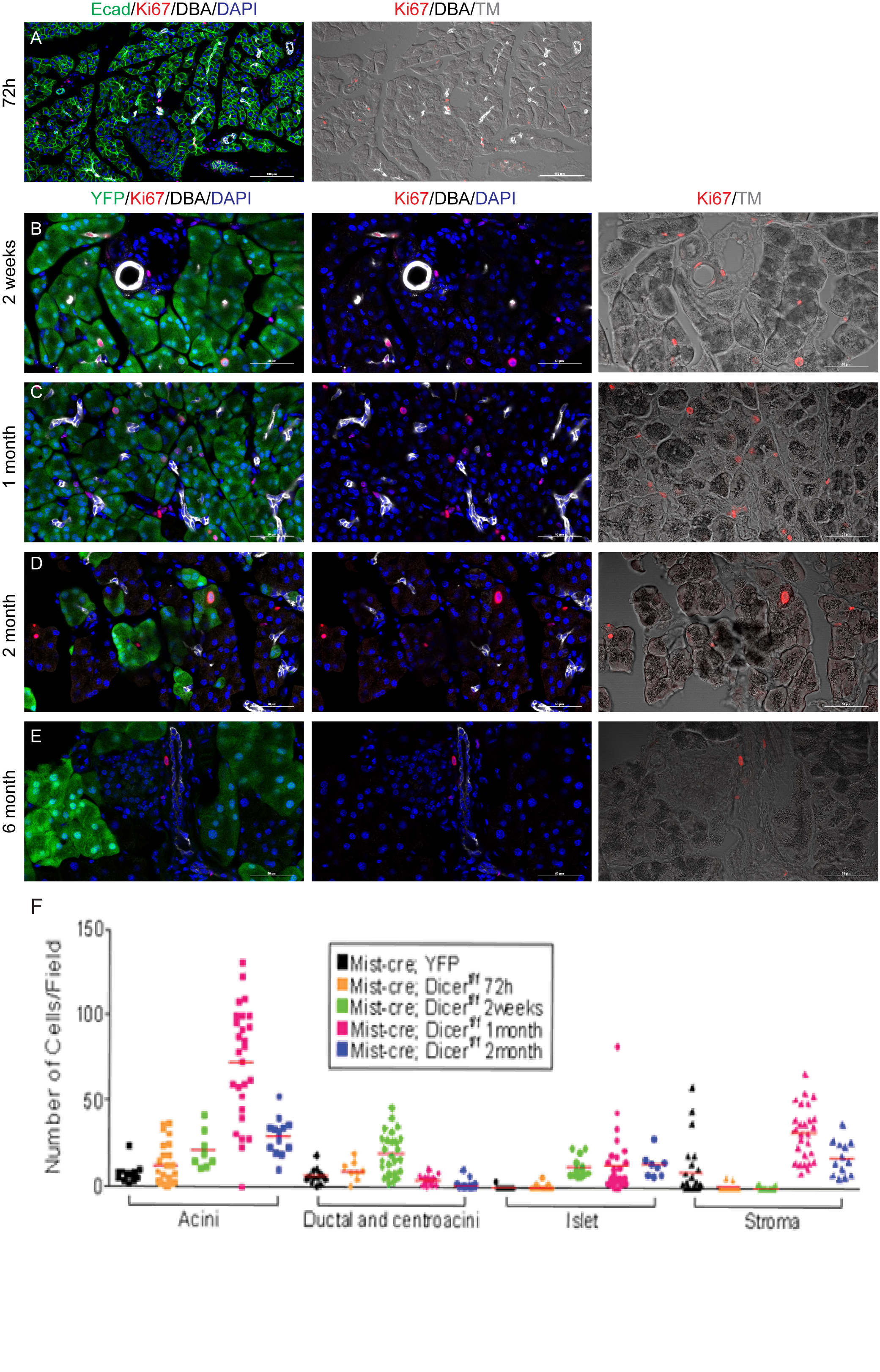

Supplement: Figure S2 — Tissue-wide proliferation response after Dicer deletion. Ki67 (red) labels proliferating cells in pancreata (A) 75 h, (B) 2 weeks, (C) 1 months, (D) 2 months, (E) 6 months post tamoxifen administration. In (A) Ecad is shown in green, DBA in white and DAPI in blue. In B–E, YFP (green) labels cell lineages in which Cre-mediated recombination has occurred. Note relative expansion of YFP-negative regions at 2 months and 6 months post-tamoxifen. Sections are co-stained with DBA (white) and DAPI (blue). Quantification of Ki67-positive cells per high power field is shown in (F). (TIF) [file pone.0113127.s002.tif]
